# Comparative transcriptome profiling reveals cold stress responsiveness in two contrasting Chinese jujube cultivars

**DOI:** 10.1186/s12870-020-02450-z

**Published:** 2020-05-27

**Authors:** Heying Zhou, Ying He, Yongsheng Zhu, Meiyu Li, Shuang Song, Wenhao Bo, Yingyue Li, Xiaoming Pang

**Affiliations:** 1 Beijing Advanced Innovation Center for Tree Breeding by Molecular Design, National Engineering Laboratory for Tree Breeding, Key Laboratory of Genetics and Breeding in Forest Trees and Ornamental Plants, Ministry of Education, College of Biological Sciences and Biotechnology, Beijing Forestry University, Beijing, 100083 China; 2 Institute of Crop, Wuhan Academy of Agricultural Sciences, Wuhan, 430074 China

**Keywords:** *Ziziphus jujuba*, ‘Dongzao’, ‘Jinsixiaozao’, Freezing stress, RNA-seq, DEGs

## Abstract

**Background:**

Low temperature is a major factor influencing the growth and development of Chinese jujube (*Ziziphus jujuba* Mill.) in cold winter and spring. Little is known about the molecular mechanisms enabling jujube to cope with different freezing stress conditions. To elucidate the freezing-related molecular mechanism, we conducted comparative transcriptome analysis between ‘Dongzao’ (low freezing tolerance cultivar) and ‘Jinsixiaozao’ (high freezing tolerance cultivar) using RNA-Seq.

**Results:**

More than 20,000 genes were detected at chilling (4 °C) and freezing (− 10 °C, − 20 °C, − 30 °C and − 40 °C) stress between the two cultivars. The numbers of differentially expressed genes (DEGs) between the two cultivars were 1831, 2030, 1993, 1845 and 2137 under the five treatments. Functional enrichment analysis suggested that the metabolic pathway, response to stimulus and catalytic activity were significantly enriched under stronger freezing stress. Among the DEGs, nine participated in the Ca^2+^ signal pathway, thirty-two were identified to participate in sucrose metabolism, and others were identified to participate in the regulation of ROS, plant hormones and antifreeze proteins. In addition, important transcription factors (*WRKY*, *AP2*/*ERF*, *NAC* and *bZIP*) participating in freezing stress were activated under different degrees of freezing stress.

**Conclusions:**

Our research first provides a more comprehensive understanding of DEGs involved in freezing stress at the transcriptome level in two *Z. jujuba* cultivars with different freezing tolerances. These results may help to elucidate the molecular mechanism of freezing tolerance in jujube and also provides new insights and candidate genes for genetically enhancing freezing stress tolerance.

## Background

Low temperature, as an abiotic stress, not only influences the geographical distribution of many important crops but also negatively impacts the productivity and quality of crops every year [1, 2]. Cold stresses are divided into chilling (0–15 °C) and freezing (< 0 °C) damage, which have a large impact on the survival and geographical distribution of plants [3]. Large amounts of plant species are grown in the tropics and subtropical regions, which are mostly chilling sensitive. Nevertheless, plants can increase their freezing tolerance via cold acclimation [4, 5].

In previous studies, researchers have revealed that cold exposure results in a variety of alterations in physiology and gene expression patterns, and thousands of genes were even identified that were up- and downregulated in response to cold, including the metabolism of carbohydrates, molecular chaperones, antifreeze proteins, signal transduction (receptor kinase, protein kinase/phosphatase, Ca^2+^ binding protein) and regulatory proteins [6–10]. In addition, transcription factors could regulate gene expression to improve yields under adverse growing conditions [7, 11]. Some TFs (*WRKYs* and *MYBs*) are involved in the plant cold response [12–14]. Overexpression of *CBF* genes conferred enhanced tolerance to cold stresses [15–23], and the overexpression of *TaCBF* genes could enhance freezing tolerance in barley [24]. In addition, hydrogen peroxide (H_2_O_2_), superoxide anion (O^2−^), and hydroxyl radical (HO·) [25–30] as ROS signals can activate redox-responsive proteins, such as protein kinases and transcription factors, such as MAPK cascade [10], and activate of ZAT12 [31]. He et al. [32] found that PeSTZ1 could enhance freezing tolerance through modulation of ROS scavenging by directly regulating *PeAPX2* in *Populus euphratica*.

Chinese jujube (*Ziziphus jujuba* Mill.) is one of the most important fruit crops in China and has also been introduced to South Korea, the United States and many other countries [33]. In recent years, possibly because global temperature is increasing, abnormal extreme weather, such as cold extremes, has been observed to increase all over the world [34, 35]. Cold stress also seriously hinders the development of the jujube industry; for example, the quality and productivity of fruits are negatively affected when exposed to low temperature. In terms of woody plants, most studies are focused on physiological and biochemical factors, and little is known about the molecular mechanisms of freezing tolerance [36, 37]. Saadati et al. [37] analyzed the freezing tolerance of seven olive cultivars by measuring some biochemical and physiological parameters. Nui et al. [38] found some candidate genes potentially involved in freezing tolerance in *Prunus persica* through high-throughput RNA sequencing. High-throughput RNA sequencing (RNA-seq) technology is useful for the comprehensive analysis of gene functions and metabolic pathways at the transcriptional level. However, it remains unclear how the transcriptome of jujubes responds to freezing stress, and important genes involved in different freezing tolerances are rarely reported. We found that ‘Jinsixiaozao’ showed a much higher freezing tolerance than ‘Dongzao’ by LT50 values, which makes them models for investigating the mechanisms of jujube freezing tolerance (Fig. S1). Thus, we used RNA-seq technology to investigate the transcriptome difference between the two cultivars under different degrees of freezing stress. This work provides useful information for understanding the molecular regulation that occurs under freezing tolerance using two cultivars with contrasting freezing tolerance, which provides a rich genetic resource for further research investigating freezing response in jujube and other woody plants.

## Results

### Quality analysis and sequence assembly of RNA-seq data

Total RNA extracted from the xylem of jujube branches was employed to build expression libraries for sequencing by using the Illumina High-Seq sequencing system. We obtained 30 library reads, and each library had approximately 89 million reads. After filtering, clean reads varied from 87 million to 88 million, which occupied on average 97.47% of the total reads of all libraries (Table 1). In all samples, the Q30 value was over 86%, and the GC content was 43%. The genome map rate accounted for more than 73%, and the gene map rate reached 72.93 to 76.32%. A total of approximately 20,000 genes were detected to be expressed in each library. In addition, more novel transcripts were detected in both DZD and JSD. Then, regarding alternative splicing, each sample had a similar number of events. Indel, JSD has the maximum mark, significantly more than in DZC. Pearson’s correlation coefficients among the three biological replicates were high (γ = 0.91–0.98).

**Table 1:** Summary of the RNA-seq data collected from ‘Dongzao’ and ‘Jinsixiaozao’

Sample	Raw data	Clean reads	Q30(%)	GC(%)	Genome map Rate	Gene map Rate	Expressed Gene	Expressed Transcripts	Novel Transcripts	Alternative Splicing	Indel
DZCK	89,647,985	87,382,161	86.91	44.19	72.64%	74.96%	20,411	25,818	988	90,011	4342
DZA	89,648,769	87,565,991	88.23	44.12	73.14%	75.11%	20,362	25,710	954	87,733	8628
DZB	89,648,625	87,947,768	87.87	44.33	73.84%	76.32%	20,431	25,881	980	88,983	5811
DZC	89,648,887	87,910,197	88.73	44.19	73.52%	75.50%	20,192	25,458	924	85,154	3927
DZD	89,649,519	87,899,251	88.22	44.02	73.98%	73.91%	21,546	26,853	1066	87,281	4230
JSCK	89,647,603	87,753,183	87.40	43.64	73.12%	74.68%	20,416	25,724	983	89,086	5555
JSA	89,648,885	87,822,119	89.13	43.69	72.43%	74.23%	20,264	25,542	983	91,659	4705
JSB	89,648,594	88,010,731	88.92	43.91	72.85%	74.63%	20,154	25,425	972	86,901	6013
JSC	89,648,221	87,657,475	87.74	43.70	72.58%	73.31%	19,871	25,011	963	87,767	5261
JSD	89,647,797	87,561,609	87.25	43.69	73.28%	72.93%	20,648	26,003	1051	99,095	6220

*DZCK* ‘Dongzao’ treated at 4 °C, *DZA* ‘Dongzao’ treated at −10 °C, *DZB* ‘Dongzao’ treated at −20 °C, *DZC* ‘Dongzao’ treated at −30 °C, *DZD* ‘Dongzao’ treated at −40 °C, *JSCK* ‘Jinsixiaozao’ treated at 4 °C, *JSA* ‘Jinsixiaozao’ treated at − 10 °C, *JSB* ‘Jinsixiaozao’ treated at − 20 °C, *JSC* ‘Jinsixiaozao’ treated at − 30 °C, *JSD* ‘Jinsixiaozao’ treated at − 40 °C

### DEGs obtained by comparing two cultivars

**Fig. 1:**
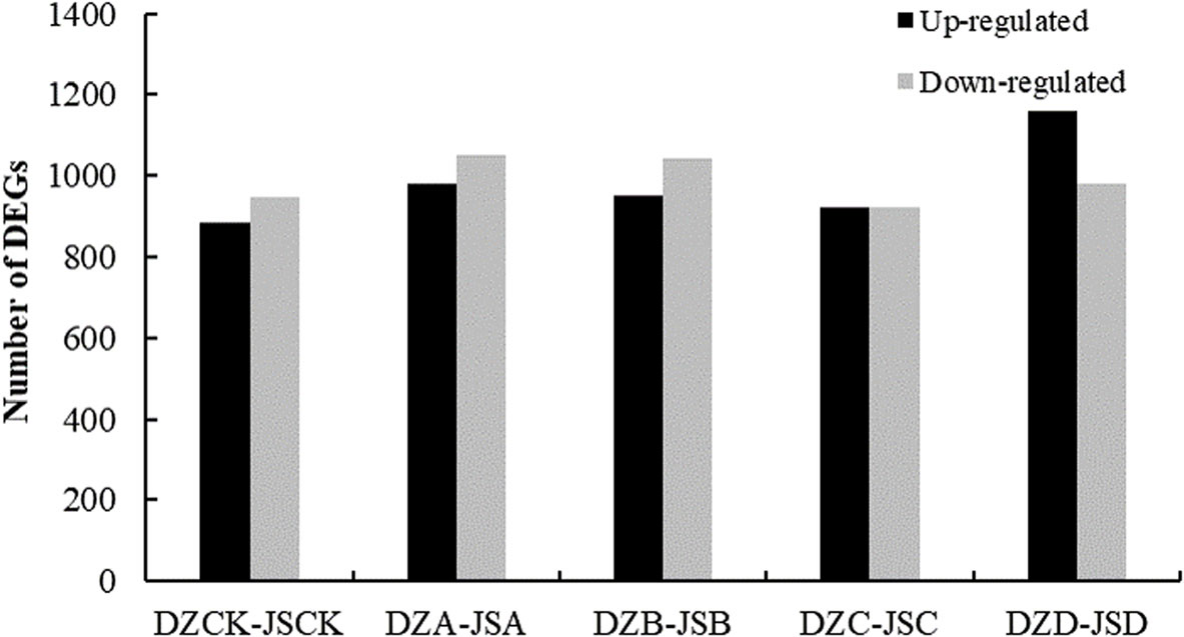
Numbers of DEGs in two cultivars at the same freezing stress. DZCK, ‘Dongzao’ treated at 4 °C; DZA: ‘Dongzao’ treated at − 10 °C; DZB: ‘Dongzao’ treated at − 20 °C; DZC: ‘Dongzao’ treated at − 30 °C; DZD: ‘Dongzao’ treated at − 40 °C; JSCK: ‘Jinsixiaozao’ treated at 4 °C; JSA: ‘Jinsixiaozao’ treated at − 10 °C; JSB: ‘Jinsixiaozao’ treated at − 20 °C; JSC: ‘Jinsixiaozao’ treated at − 30 °C; JSD: ‘Jinsixiaozao’ treated at − 40 °C

To understand the differences between different cultivars in response to freezing stress, gene expression profiles in ‘Dongzao’ and ‘Jinsixiaozao’ at the same level of freezing stress were further analyzed though FPKM values. The detailed statistics are shown in Additional file 2. We investigated DEGs specific to each freezing stress by comparing ‘Dongzao’ to ‘Jinsixiaozao’. There were 1831 (885 upregulated genes and 946 downregulated genes), 2030 (979 upregulated genes and 1051 downregulated genes), 1993 (949 upregulated and 1044 downregulated genes), 1845 (923 upregulated and 922 downregulated genes) and 2137 (1158 upregulated and 979 downregulated genes) DEGs were identified at 4 °C, − 10 °C, − 20 °C, − 30 °C and − 40 °C, respectively, suggesting the responsiveness of these genes to freezing and cold resistance (Fig. 1 and Additional file 3).

**Fig. 2:**
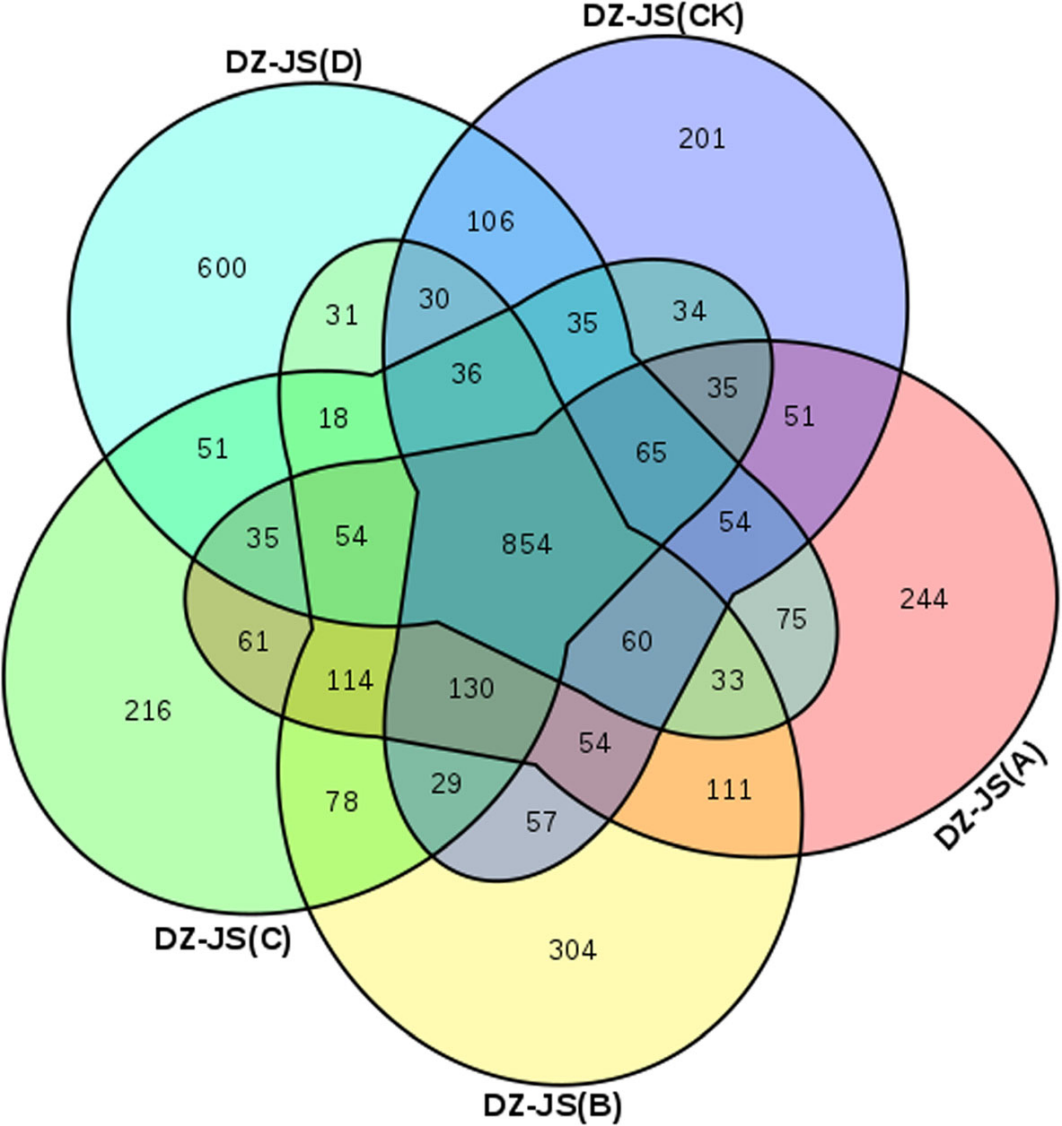
Venn diagram of DEGs under the same freezing stress in ‘Dongzao’ and ‘Jinsixiaozao’.DZ, ‘Dongzao’; JS, ‘Jinsixiaozao’; CK, treated at 4 °C; A, treated at − 10 °C; B, treated at − 20 °C; C, treated at − 30 °C; D, treated at − 40 °C

In five different degrees of cold stress, as shown in Fig. 2, 854 common DEGs were detected in both cultivars, which may suggest that these DEGs are involved in the process of dealing with low temperature under different low temperatures, and both participate in the same pathways in response to freezing stress. In addition, at − 40 °C, the number of unique DEGs (600) was significantly higher than that in the other groups, indicating that these genes may be related to the difference in cold tolerance between ‘Dongzao’ and ‘Jinsixiaozao’.

In this study, total RNA from branches was used for qRT-PCR validation. As shown in Fig. S2, six DEGs showed similar expression pattern between the qRT-PCR data and the RNA-seq results, demonstrating that the RNA-seq data is highly reliable for further analysis.

### Function annotation and enrichment analysis of DEGs

**Fig. 3:**
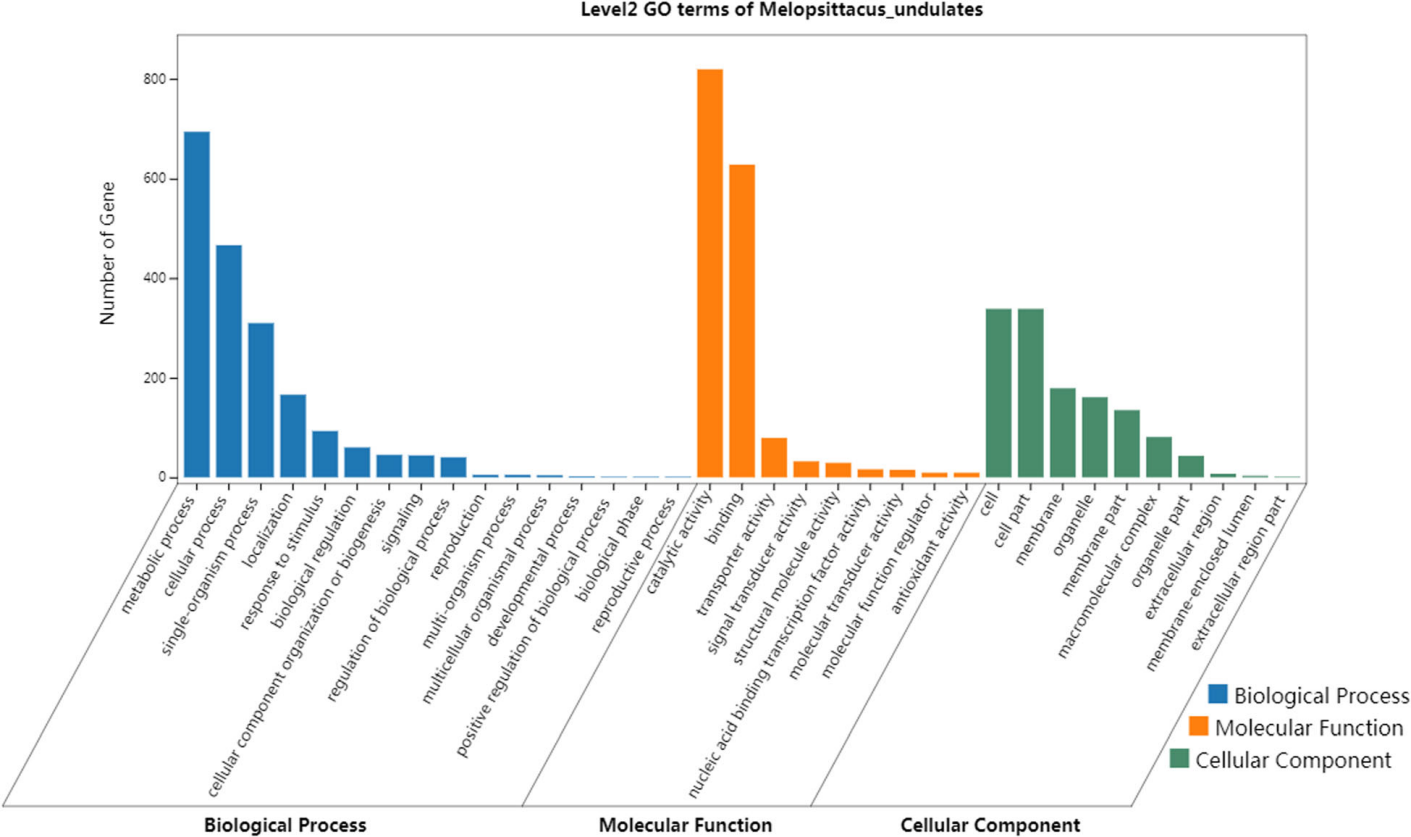
GO analysis of DEGs at the same freezing stress between two cultivars. GO classification of DEGs under cold stress in ‘Dongzao’ and ‘Jinsixiaozao’, respectively. The Y and X axes correspond to GO terms and the number of DEGs

To understand the functions of the discovered DEGs between the two cultivars, all DEGs were searched against the protein database, followed by Gene Ontology (GO) analysis to assess the putative functions of the genes. Among the GO terms of each treatment, the stronger freezing stress had a larger proportion of DEGs, and the more metabolic processes were modified and changed (Fig. 3). In the comparative analysis of five groups, the DEGs of these GO terms were classified into ‘metabolic process’, ‘cellular process’ and ‘response to stimulus’; and ‘cell’, ‘cell part’ and ‘membrane’ in cell components; ‘catalytic activity’, ‘binding’, ‘transporter activity’ in molecular function.

**Fig. 4:**
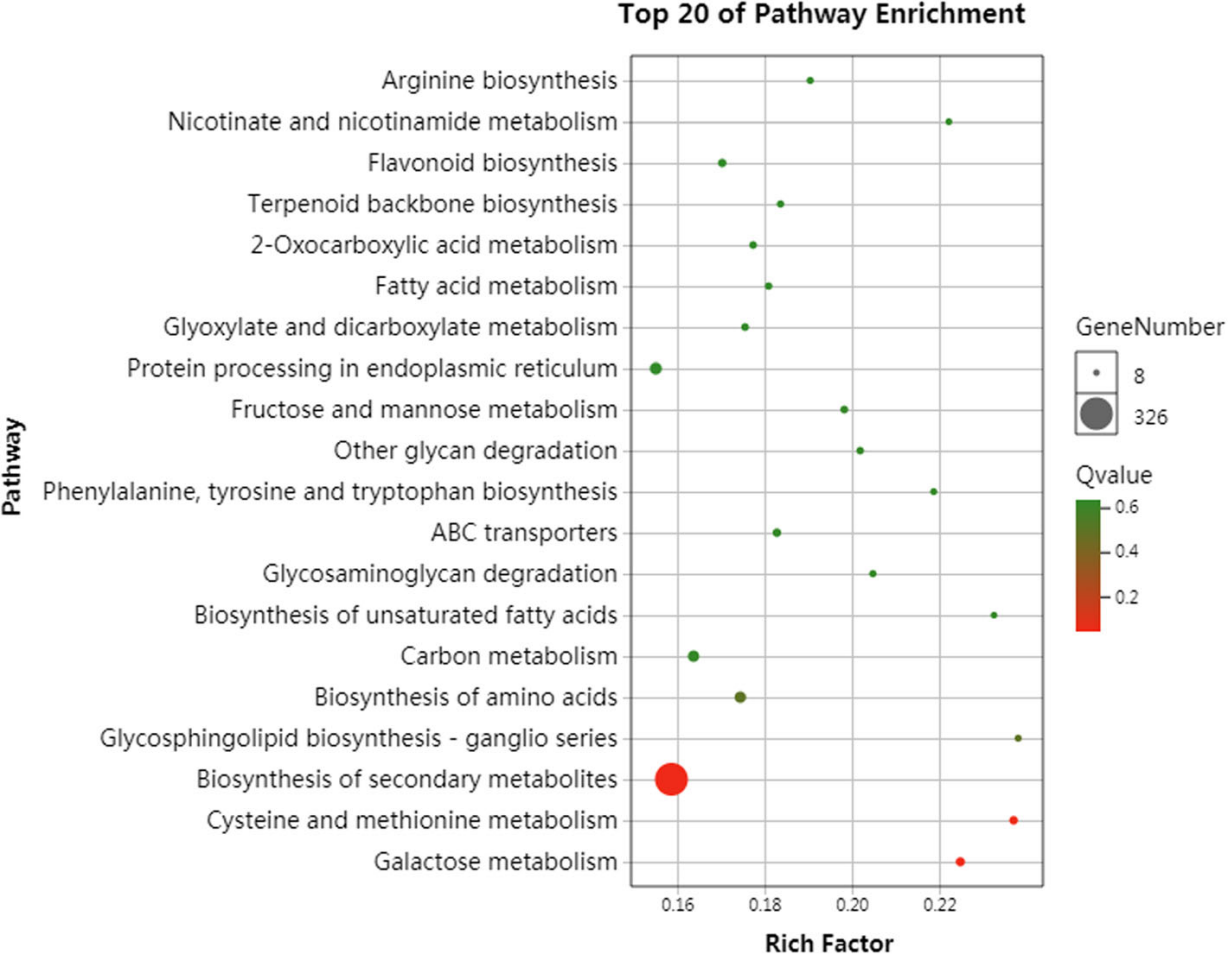
KEGG enrichment analysis of DEGs at the same freezing stress between two cultivars. KEGG pathway classifcation of DEGs under cold stress in ‘Dongzao’ and ‘Jinsixiaozao’, respectively. The Y axis corresponds to KEGG pathway, the X axis shows the enrichment ratio between the number of DEGs enriched in a particular pathway. The color of the dot represents q-value, and the size of the dot represents the number of DEGs mapped to the referent pathway

In addition, to elucidate the metabolic pathways involved in freezing stress, 20 common metabolic processes were most enriched at the five freezing treatments in the two cultivars (Fig. 4). Among these top pathways, the carbohydrate metabolism pathway, galactose metabolism, fructose and mannose metabolism were associated with many up/downregulated genes. Furthermore, in the comparison of the five groups, there was some pathway enrichment in the stronger freezing stress in ‘Jinsixiaozao’, such as peroxide of metabolic pathways, which was only significantly enriched at − 30 °C, and the ABC transporter process was enriched at − 40 °C. In brief, many genes of different metabolic pathways were involved in the regulation of freezing stress in jujube, and further study on the differential expression pattern in these pathways has great significance for revealing the cold resistance of jujube.

To further analyze the difference in jujube at extreme temperatures, we conducted a comparative analysis of the two cultivars with temperatures of − 30 °C and − 40 °C. In comparison, we found that more significantly DEGs were found in the stronger freezing stress. The peroxisome pathway was enriched in DZC vs. JSC, and the ABC transporters pathway was enriched in DZD vs. JSD. In addition, 107,430,353, annotated to the *WRKY* family, was significantly changed at lower temperatures. In the ABC family, some members (such as 107,429,561, 107,408,533, 107,404,223, 107,414,655, and 107,416,457) simultaneously exhibited significant upregulation. The ratio of DEGs related to kinase activity and carbohydrate metabolism also increased under the stronger freezing stress than under weak freezing stress.

### Comparison analysis at different degrees of freezing stress in ‘Dongzao’ and ‘Jinsixiaozao’

**Fig. 5:**
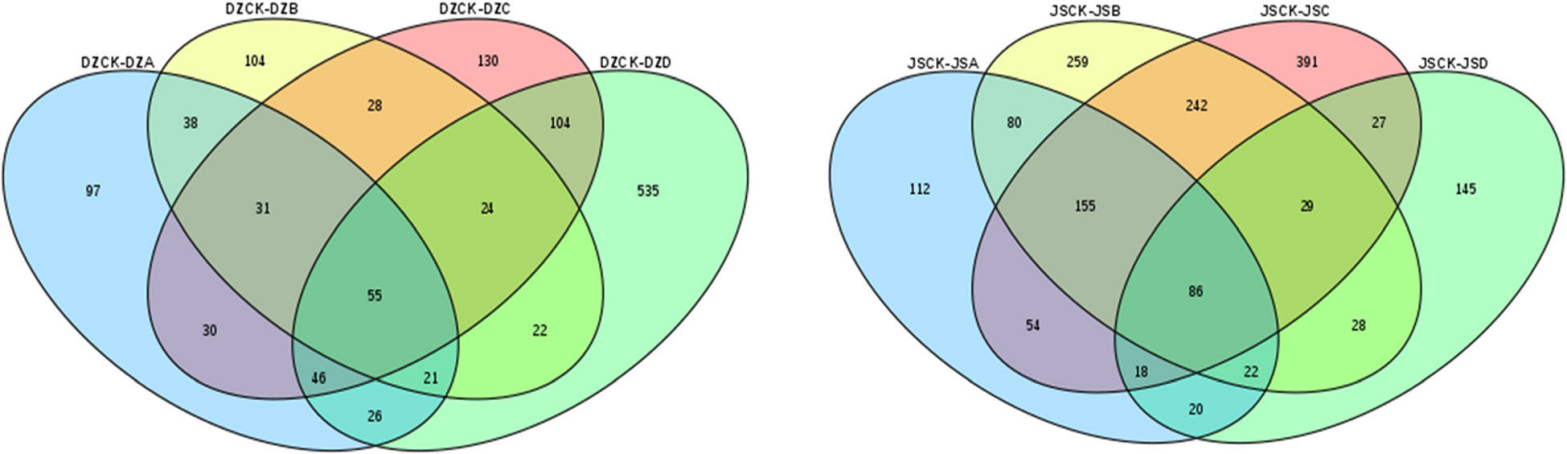
Venn diagram of DEGs in ‘Dongzao’ (left) and ‘Jinsixiaozao’ (right). DZCK, ‘Dongzao’ treated at 4 °C; DZA: ‘Dongzao’ treated at − 10 °C; DZB: ‘Dongzao’ treated at − 20 °C; DZC: ‘Dongzao’ treated at − 30 °C; DZD: ‘Dongzao’ treated at − 40 °C; JSCK: ‘Jinsixiaozao’ treated at 4 °C; JSA: ‘Jinsixiaozao’ treated at − 10 °C; JSB: ‘Jinsixiaozao’ treated at − 20 °C; JSC: ‘Jinsixiaozao’ treated at − 30 °C; JSD: ‘Jinsixiaozao’ treated at − 40 °C

To understand the different effects on jujube at different degrees of freezing stress, we also compared four freezing stresses in ‘Dongzao’ and ‘Jinsixiaozao’. In ‘Dongzao’, DZCK was used as a control, and 1291 DEGs were identified at different degrees of freezing stress, although only 55 common DEGs were identified in all freezing stresses (Fig. 5, Fig. S3 and Additional file 4). Then, these DEGs were further screened and 327 significant DEGs (PPEE< 0.05 and |Log2| ≥ 1) were identified. Of these significant DEGs, some genes were related to the Ca^2+^ signaling pathway, including upregulated gene (107413690) and downregulated genes (107,404,917, 107,423,107, 107,425,924, 107,417,689, 107,421,107, 107,422,102, 107,430,853 and 107,424,939). Some DEGs related to sucrose metabolism were classified into biological processes among the four groups, including 7 upregulated and 25 downregulated. Furthermore, regarding transcription factors, through the analysis of DEGs, we found that the genes belonging to the *NAC*, *WRKY*, *MYB*, *AP2*/*ERF* and *bHLH* families all showed a significant up- or downregulation. A gene (107424282) homologous to the grape *MYB* transcription factor is significantly downregulated under cold stress, suggesting that it may negatively regulate the cold resistance of jujube trees. In addition, the *NAC* transcription factor (107435293) had a higher fold change at − 30 °C, indicating that this transcription factor may regulate the cold resistance of jujube trees in response to freezing stress.

In the comparison of four groups of ‘Jinsixiaozao’, JSCK as a control, a total of 1668 DEGs were identified (Fig. 5, Fig. S4 and Additional file 5), and 86 common DEGs were present in all comparisons. Then we focused analysis on the significant DEGs (PPEE< 0.05 and |Log2| ≥ 1). A total of 71 significantly DEGs were identified at − 10 °C, 39 genes were upregulated and 32 were downregulated. In the case of − 20 °C, 108 DEGs were identified, including 31 upregulated genes and 77 downregulated genes. With decreasing temperature, 31 genes were upregulated and 70 genes were downregulated at, − 30 °C. At − 40 °C, only 43 genes had larger changes. Similar to the results of ‘Dongzao’, many genes associated with abiotic stress have been annotated in ‘Jinsixiaozao’, such as sucrose metabolism and the Ca^2+^ signal pathway. Interestingly, among these DEGs, 107,422,102 was significantly downregulated at − 10 °C in ‘Jinsixiaozao’, while it was not changed until the temperature dropped to − 40 °C in ‘Dongzao’. This finding may be due to the different regulatory mechanisms of the two cultivars in response to freezing stress, thereby reflecting different cold resistance.

### Key transcription factors associated with freezing stress

**Fig. 6:**
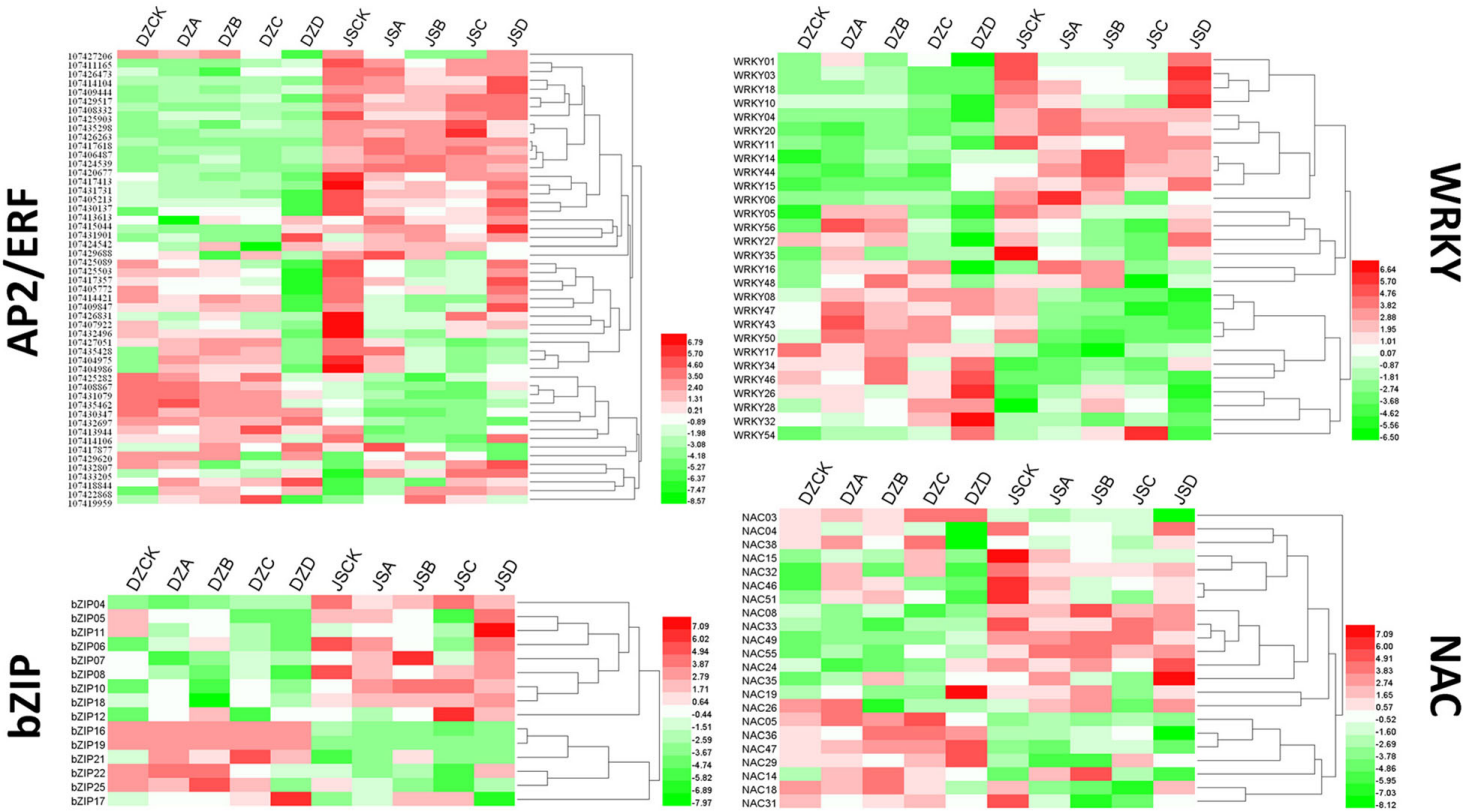
Expression profiles of the jujube *AP2*/*ERF*, *WRKY*, *bZIP* and *NAC* genes in two cultivars. DZCK, ‘Dongzao’ treated at 4 °C; DZA: ‘Dongzao’ treated at − 10 °C; DZB: ‘Dongzao’ treated at − 20 °C; DZC: ‘Dongzao’ treated at − 30 °C; DZD: ‘Dongzao’ treated at − 40 °C; JSCK: ‘Jinsixiaozao’ treated at 4 °C; JSA: ‘Jinsixiaozao’ treated at − 10 °C; JSB: ‘Jinsixiaozao’ treated at − 20 °C; JSC: ‘Jinsixiaozao’ treated at − 30 °C; JSD: ‘Jinsixiaozao’ treated at − 40 °C.Data were normalized based on the mean expression value of each gene in all samples analysed. Red and green boxes indicate high and low expression levels, respectively, for each gene

In this study, we further analyzed the expression of key transcription factors (*AP2*/*ERF*, *WRKY*, *NAC* and *bZIP*), which have been reported to be involved in freezing stress. All FPKM values of these genes are listed in Additional file 6. In all libraries, we identified a total of 28 *WRKY* members, 52 *AP2*/*ERF* members, 22 *NAC* members and 15 *bZIP* members in two cultivars (Fig. 6). Comparison of the expression of these TFs between the two cultivars identified *WRKY* members; some members were highly expressed in ‘Dongzao’, and others were highly expressed in ‘Jinsixiaozao’. Among the TFs, the expression levels of *WRKY04* (107417519), *WRKY11* (107414569), and *WRKY20* (107419756) in ‘Jinsixiaozao’ were completely different from those in ‘Dongzao’. In the *AP2/ERF* gene family, three of 52 *AP2*/*ERF* members (107,418,844, 107,432,807 and 107,422,868) were upregulated under light cold stress in ‘Dongzao’ but began to change under the lower temperature ‘Jinsixiaozao’. In terms of *NAC* and *bZIP*, *NAC19* had a higher expression level in DZD than in other samples, while in ‘Jinsixiaozao’, the expression level was higher at all five temperatures; *bZIP* members are generally divided into two expression patterns. The observed expression differences of these transcription factors between the two cultivars suggest that TFs may play important roles in the response to different freezing stresses in jujube.

### Key DEGs in the galactose metabolism pathway

The accumulation of stachyose plays an important role in plant resistance to low temperature stress, and α-galactosidase is important in the decomposition process of stachyose. In the comparison of the five groups, the galactose metabolic pathway was significantly enriched under each temperature condition. Thus, we further characterized the identified DEGs involved in the metabolism of galactose. During the metabolism of galactose, some genes associated with the galactose, stachyose, raffinose, and sucrose pathways showed significant differences in coping with freezing stress. As listed in Table S2, among these genes, 107,411,641 is a key gene in the process of UDP-galactose, which is significantly upregulated under stronger freezing stress, which will enhance the synthesis pathway of stachyose. However, in the pathway of stachyose decomposition into galactose and raffinose, the FPKM value of genes (107,425,264, 107,430,302, 107,414,011 and 107,428,511) decreased, which reduced the decomposition of stachyose (Fig. S5).

## Discussion

Over the past years, extreme weather has occurred frequently, and the variations in climate have affected the growth and development of jujubes and even caused their death. Multiple responses to cold stress have been reported in many plant species, reflected by variations in transcription or metabolism [39]. Recently, significant advances have been made regarding the understanding of plant cold-signaling perception and transduction; however, we are still far from fully understanding the molecular mechanism under cold signal perception and transduction in plants [40–42]. In our study, the comparison of transcriptome profiles between ‘Jinsixiaozao’ and ‘Dongzao’ was reported to identify the genes responsible for freezing resistance. We first provided a more comprehensive analysis of changes in important metabolic pathways and transcription factors in jujube. Our research could also provide a reference for other woody plants and facilitate breeding for the future development of improved cold- tolerance in jujube cultivars.

### Identification of DEGs in two cultivars

In two cultivars, some DEGs were investigated and hundreds of freezing-related genes were identified in 30 libraries of jujube branches (approximately 87 million reads in ‘Dongzao’ and ‘Jinsixiaozao’). A total of 362 significantly DEGs (323 DEGs in ‘Jinsixiaozao’) were found to be differentially expressed in ‘Dongzao’ at different degrees of freezing stress. Compared with the control, most significant DEGs were downregulated. This result is similar to Kou et al. [43] in potato, which suggested that different cultivars have similar responses to cold resistance. However, this finding is different from that of Niu et al.’s [38] study in *Prunus persica* suffering from freezing tolerance, in which the number of upregulated genes were greater than that of downregulated genes. Moreover, GO classifications of ‘Dongzao’ and ‘Jinsixiaozao’ were compared and enriched in functional categories, mainly ‘catalytic activity’, ‘metabolic process’, ‘cell part’ and ‘binding’. KEGG pathway analysis revealed that the principal enriched pathways comprised plant hormone signal transduction, metabolic pathways, ribosome and secondary metabolic pathways. With the data provided by the comprehensive analysis two contrasting Chinese jujube cultivars, more important DEGs involved in freezing stress were characterized, which can further contribute to understanding *Z. jujuba*.

### Physiological response-related genes under freezing stress

Some research shows that plants could increase their tolerance by synthesizing numerous protective substances and proteins under these conditions [44–47]. Raffinose family oligosaccharides (RFOs) are a class of functional oligosaccharides unique to plants, mainly raffinose, stachyose and verbascose. Some studies have found that RFO can improve the ability of plants to cope with adversity [46, 48]. The more RFOs accumulate, the stronger the ability of plants to be exposed to low temperatures becomes. Galactose and related enzymes are involved in the RFO metabolic pathway [48]. In our study, galactose metabolism was significantly enriched, and relative genes in this pathway were up- or downregulated to cope with cold stress. Among these genes, 107,426,117 (stachyose synthetase), 107,415,484 (raffinose synthase) and 107,411,641 (inositol 3-alpha-galactosyltransferase) were significantly upregulated under the stronger freezing treatment in ‘Jinsixiaozao’, but some of them did not significantly change under the weak freezing stress. This finding may suggest these genes play a more important role in galactose metabolism in response to cold, especially under stronger freezing stresses. In addition, late embryogenesis abundant protein (LEA) could respond to abiotic stresses such as low temperature and drought [49]. In our study, the upregulation of the *LEA* gene (107409127) indicates that *LEA* genes are involved in freezing tolerance in ‘Jinsixiaozao’ (− 40 °C). The function of LEA protein in this study should be further studied as a regulatory mechanism of freezing response.

### Cold signal transduction related genes under freezing stress

The signal transduction pathway is known to play crucial roles in the response to freezing stress; among these, Ca^2+^ is an important second messenger in plants [2, 50]. The enriched Ca^2+^ signaling pathway genes play critical roles in signaling pathways related to the response to freezing stress [38]. In our research, 34 DEGs were identified as related to calcium-binding proteins, calmodulin and Ca^2+^ signals. Among them, the gene (107413690) showed a significant upregulation in ‘Dongzao’, indicating that these genes play important roles in jujube resistance to freezing stress and the function of these genes should be further studied.

In addition to Ca^2+^, ROS also play important roles [51]. On the one hand, they induce gene expression and protein synthesis to protect cells from stress; on the other hand, they induce oxidative stress [52, 53]. ROS can also directly stimulate the expression of genes in activating stress-related pathways, such as the MAPK cascade pathway, hormone signaling pathway and calcium signaling pathway. In our study, some genes with antioxidant activity were involved in freezing stress. Among these DEGs, six genes (one upregulated gene and five downregulated genes) were involved in redox signaling, such as gene 107,403,552, which is differentially expressed under four degrees of freezing stress in ‘Jinsixiaozao’, indicating that they played different roles in cold signal transduction under freezing stress.

### Transcriptional regulation-related genes under freezing stress

Transcription factors play various important functions in plant abiotic stress tolerance. Some studies have shown that most cold-regulated transcription factor genes are impaired in response to cold stress in *Arabidopsis* [54–56]. Many studies have shown that some transcription factors play important roles in plant freezing tolerance [57–59]. For example, the *AP2*/*ERF* factors *RAP2*.1 and *RAP2*.6 and the C_2_H_2_-type zinc finger *STZ*/*ZAT10* belong to the *CBF* regulon [60, 61]. Furthermore, *NAC* was also shown to enhance expression during loquat fruit chilling; however, it could not bind directly to promoters of lignin biosynthesis genes [62]. Many MYB-type transcription factors, such as *AtMYB85* [63] and *AtMYB88* [64, 65], increase cold hardiness by CBF-dependent and CBF-independent pathways in apple. In other species, *OsMYB4* transgenic lines of *Arabidopsis* exhibited improved chilling and freezing tolerance with a dwarf phenotype [66]. In this study, the expression patterns of the *AP2*/*ERF*, *WRKY*, *NAC* and *bZIP* gene families were analyzed, and we found that different members play different roles in the two cultivars. For example, in the *AP2*/*ERF* family, different members of the family are involved in the response to low temperatures. Among these genes, 107,414,104 expression showed significant changes at − 10 °C; genes 107,432,697 and 107,433,390 were at − 20 °C; 107,426,263 and 107,433,390 were at − 30 °C; however, 107,414,104, 107,432,697 and 107,433,390 changed significantly at the lowest temperatures. Similarly, other TF members are generally divided into two expression patterns. These differences suggest that the two cultivars have different responses to cold stress, even for different degrees of freezing stress. We did not go further to verify the function of these genes but listed the significant differences in this work. Thus, the emphasis of future research could focus on the role of these genes in metabolic pathways and understand the regulatory mechanism of genes.

In addition, some studies have found that alternative splicing (AS) events are important post-transcriptional regulators and are required for reprogramming gene expression under environmental stress. Previous studies showed that the number of AS events was significantly higher compared to control plants when plants were exposed to different stresses, especially low temperatures [67]. To analyze the variation in AS under cold stress, in this study, we detected that all genes containing introns were alternately spliced under cold stress (Fig. S6). On the one hand, compared with the control, the number of AS events increases slightly as the temperature drops, and we hypothesize that plants can fine-tune gene expression by AS events in response to temperature changes. On the other hand, most AS genes were not differentially regulated by stress, suggesting that AS may represent an independent gene regulatory layer that responds to cold stress.

### Post-translational regulation-related genes under freezing stress

In addition, post-translational modifications are also important for plant cold responses. Dong et al. [68] found that HOS1 can degrade ICE1 and negatively regulate CBFs and downstream cold response genes by ubiquitination under low temperature stress. In this study, the homologous gene (107429115) was found to be significantly upregulated. In addition, with the enhancement of freezing stress, the pathways of phenylpropanoid biosynthesis and flavonoid biosynthesis were also strengthened. Among the significantly DEGs, genes (107,429,115 and 107,415,362) are glycosyl-transferase-related genes, and the former is significantly upregulated at − 40 °C, and the latter is downregulated, which may mean that they are positively and negatively regulated factors in the process of regulating the cold resistance of jujube, which are involved in different regulatory signaling pathways. Moreover, MLO proteins are a unique protein family in plants, and the expression of many MLO genes is affected by various biotic and abiotic stresses [69]. There is a calmodulin binding region at the C-terminus of the MLO protein, which allows the MLO protein to bind to calmodulin as a second messenger to transmit signals to the downstream pathway. The CaM protein binds to the CaMBD of the MLO protein to increase the activity of the MLO protein. In our data, we found that the gene 107,409,553 encoding the MLO protein showed a significant upregulation when ‘Jinsixiaozao’ was at − 40 °C but showed downregulation in ‘Dongzao’. This finding may indicate that the genes regulated by calcium are varietally different between the two cultivars in response to freezing stress.

Some studies have shown that cold stress can restrict plant growth and development at different levels, for example the cell and tissue levels. Roots and branches are both the tissues that predominantly perceive and transduce cold signal in cold stress responses [38, 40], we comprehensively analyzed the jujube branches at the tissue level which provided valuable information and genetic resources for improving cold stress in jujube. In addition, we found some significant DEGs under higher degrees of freezing stress, which may suggest that the two cultivars have different responses to different degrees of freezing stress in the metabolic pathway and biosynthesis of galactose. Jujubes sense freezing stress through membrane rigidification and cellular changes, leading to an influx of Ca^2+^, reactive oxygen species (ROS) and phytohormone changed. The signaling pathways activate protein kinases and changed various downstream transcription factors family proteins, such as WRKY, AP2/ERF, NAC and bZIP. The activation of TFs triggers the expression of downstream responsive genes to the stress treatment, as well as the induction of biochemical responses leading to freezing tolerance. These results may provide evidence to support that ‘Jinsixiaozao’ has much higher freezing tolerance than ‘Dongzao’. Our understanding of the regulatory mechanisms may provide a reference for further research investigating the role of different tissues in freezing stress and other abiotic stresses.

## Conclusions

Our present study first provides a comprehensive analysis via transcriptome sequencing between two Chinese jujube cultivars (‘Dongzao’ and ‘Jinsixiaozao’) under freezing stress. The important genes involved in signal transduction and transcriptional and post-translational regulation were differentially expressed between these two cultivars. Some different members of the *AP2/ERF*, *WRKY*, *NAC* and *bZIP* gene families were significantly up/downregulated in the two cultivars. In addition, some DEGs, such as 107,426,117 and 107,411,641, involved in galactose metabolism were significantly upregulated under higher degree freezing treatment in ‘Jinsixiaozao’ than in ‘Dongzao’, which further highlights the importance of sugar metabolism involved in freezing tolerance in plants. The present results may explain why ‘Jinsixiaozao’ has considerably higher freezing tolerance than ‘Dongzao’ and may elucidate the molecular mechanisms of freezing tolerance. The important DEGs discovered in this study may facilitate further study on freezing tolerance mechanisms in jujube and other woody plants.

## Methods

### Plant materials and cold treatment

The current year’s branches of cold-sensitive cultivar ‘Dongzao’ (DZ) and cold-tolerant cultivar ‘Jinsixiaozao’ (JS) were collected from the National Key Base for Improved Chinese Jujube Cultivar (Cangzhou, China). The collected branches were placed at 4 °C for 10 h and used as controls (DZCK and JSCK), the others were treated and maintain 10 h at the freezing temperature: -10 °C (DZA and JSA), − 20 °C (DZB and JSB), − 30 °C (DZC and JSC) and − 40 °C (DZD and JSD). Then the xylem of the branches was collected and snap-frozen in liquid nitrogen. Three replicates were used for each treatment.

### RNA-seq library construction and sequencing

Total RNA were extracted from the 30 samples using the Plant RNA Kit (Omega) according to the manufacturer’s instructions. Three biologic replicates for each treatment were used for analyzing transcriptome. The library products were ready for sequencing analysis using an Illumina Genome Analyzer at Beijing Genomics Institute (Shenzhen, China) in 2018. Raw data of fastq format were firstly processed through in-house perl scripts to obtain clean data by removing the reads containing adapter, ploy-N and low-quality reads. All the subsequent analyses were based on the clean data. Reference genome and gene model annotation files were based on *Ziziphus jujuba* (assembly ZizJuj_1.1) on NCBI. The Z-score method using the *p*-value as a statistical significance index [70] was applied to identify differentially expressed genes.

### Identification of DEGs, GO and KEGG enrichment analysis

A cluster analysis was performed according to Eisen et al. [71]: the log2 of TPM for each gene was used for the hierarchical clustering analysis. Gene Ontology (GO) categorization was classified into molecular function, biological process, and cellular component. We used KOBAS software to test the statistical enrichment of differential expression genes in KEGG pathways (http://www.genome.jp/kegg/). Four types of AS events including exon skipping, intron retention, alternative 5′ splice site and alternative 3′ splice site were identified using TopHat [72].

### Real-time quantitative PCR

Six randomly selected DEGs were chosen for validation by real-time quantitative PCR (qRT-PCR). The sequences of the primer pairs (designed using Beacon Designer 7.2) were listed in Table S1. Real-time PCR was performed in the presence of TB Green Premix Ex Taq II (Tli RNaseH Plus) (TaKaRa, Dalian, China) and read on an IQ5 real-time PCR instrument (Bio-Rad, Hercules, CA, USA). The amplification was performed as previously reported [73], and all the experiments were performed in biological triplicate. Transcript levels were normalized against the average expression of the *Zjactin* gene (GenBank: EU916201).

## Supplementary information

**Additional file 1: Figure S1-S6 and Table S1-S2. Figure S1.** The semi-lethal temperature (LT50, °C) of different cultivars after cold acclimation. **Figure S2**. Validation by qRT-PCR of DEGs isolated from the different samples in ‘Dongzao’ and ‘Jinsixiaozao’. **Figure S3.** GO analysis of DEGs under different degree cold stress in ‘Dongzao’. The X and Y axes correspond to GO terms and the number of DEGs. **Figure S4.** GO analysis of DEGs under different degree cold stress in ‘Jinsixiaozao’. The X and Y axes correspond to GO terms and the number of DEGs. **Figure S5.** DEGs in galactose metabolism. Different boxes represent different genes in galactose metabolism. The red boxes represent up-regulated genes, and the green boxes represents down-regulated genes. **Figure S6.** Alternative Splicing Event and gene number at the same degree cold stress between two cultivars. **Table S1.** Primers for qRT-PCR. **Table S2.** DEGs involved in galactose metabolism pathways.

**Additional file 2.** FPKM values in ‘Dongzao’ and ‘Jinsixiaozao’.

**Additional file 3.** DEGs between ‘Dongzao’ and ‘Jinsixiaozao’ at the same freezing stress.

**Additional file 4.** DEGs at different degree freezing stress in ‘Dongzao’.

**Additional file 5.** DEGs at different degree freezing stress in ‘Jinsixiaozao’.

**Additional file 6.** FPKM values of Key transcription factors.

## Data Availability

The datasets used and/or analysed have been deposited in NCBI under BioProject ID: PRJNA624182.

## Abbreviations

DEGs: Differentially Expressed Genes
qRT-PCR: Quantitative real-time PCR
TF: Transcription Factor
MAPK: Mitogen-Activated Protein Kinase
FPKM: Fragments Per Kilobase Per Million Mapped Reads
AS: Alternative Splicing

## References

[1] Zhu JK (2016). Abiotic stress signaling and responses in plants. Cell..

[2] Guo XY, Liu DF, Chong K (2018). Cold signaling in plants: insights into mechanisms and regulation. J Integrative Plant Biol..

[3] Jan N, Andrabi KI (2009). Cold resistance in plants: a mystery unresolved. Electron J Biotechnol.

[4] Jia YX, Ding YL, Shi YT, Zhang XY, Gong ZZ, Yang SH (2016). The *cbfs* triple mutants reveal the essential functions of CBFs in cold acclimation and allow the definition of CBF regulons in *Arabidopsis*. New Phytol.

[5] Chinnusamy V, Zhu J, Zhu JK (2007). Cold stress regulation of gene expression in plants trends. Plant Sci.

[6] Thomashow MF (1999). Plant cold acclimation: freezing tolerance genes and regulatory mechanisms. Annu Rev Plant Physiol Plant Mol Biol.

[7] Gilmour SJ, Fowler SG, Thomashow MF (2004). *Arabidopsis* transcriptional activators CBF1, CBF2, and CBF3 have matching functional activities. Plant Mol Biol.

[8] Chinnusamy V, Schumaker K, Zhu JK (2004). Molecular genetic perspectives on cross-talk and specificity in abiotic stress signalling in plants. J Exp Bot.

[9] Min K, Chen KT, Arora R (2020). A metabolomics study of ascorbic acid-induced in situ freezing tolerance in spinach (*Spinacia oleracea* L.). Plant Direct.

[10] Colcombet J, Hirt H (2008). *Arabidopsis* MAPKs: a complex signalling network involved in multiple biological processes. Biochem J.

[11] Winfield M, Lu C, Wilson I, Coghill J, Edwards K (2010). Plant responses to cold: transcriptome analysis of wheat. Plant Biotechnol J.

[12] Mare C, Mazzucotelli E, Crosatti C, Francia E, Stanca AM, Cattivelli L (2004). HvWRKY38: a new transcription factor involved in cold- and drought response in barley. Plant Mol Biol.

[13] An JP, Li R, Qu FJ, You CX, Wang XF, Hao YJ (2018). R2R3-MYB transcription factor MdMYB23 is involved in the cold tolerance and proanthocyanidin accumulation in apple. Plant J.

[14] An Jian‐Ping, Wang Xiao‐Fei, Zhang Xiao‐Wei, Xu Hai‐Feng, Bi Si‐Qi, You Chun‐Xiang, Hao Yu‐Jin (2019). An apple MYB transcription factor regulates cold tolerance and anthocyanin accumulation and undergoes MIEL1‐mediated degradation. Plant Biotechnology Journal.

[15] Jiang BC, Shi YT, Zhang XY, Xin XY, Qi LJ, Guo HW, Li JG, Yang SH (2017). PIF3 is a negative regulator of the CBF pathway and freezing tolerance in *Arabidopsis*. Proc Natl Acad Sci.

[16] Liu ZY, Jia YX, Ding YL, Shi YT, Li Z, Guo Y, Gong ZZ, Yang SH (2017). Plasma membrane CRPK1-mediated phosphorylation of 14-3-3 proteins induces their nuclear import to fine-tune CBF signaling during cold response. Mol Cell.

[17] Fiust Anna, Rapacz Marcin (2020). Downregulation of three novel candidate genes is important for freezing tolerance of field and laboratory cold acclimated barley. Journal of Plant Physiology.

[18] Dubouzet JG, Sakuma Y, Ito Y, Kasuga M, Dubouzet EG, Miura S, Seki M, Shinozaki K, Yamaguchi-Shinozaki K (2003). OsDREB genes in rice, *Oryza sativa* L, encode transcription activators that function in drought-, high-salt- and cold-responsive gene expression. Plant J.

[19] Ma Y, Dai X, Xu Y, Luo W, Zheng X, Zeng D, Pan Y, Lin X, Liu H, Zhang D, Xiao J, Guo X, Xu S, Niu Y, Jin J, Zhang H, Xu X, Li L, Wang W, Qian Q, Ge S, Chong K (2015). COLD1 confers chilling tolerance in rice. Cell..

[20] Shen YG, Zhang WK, He SJ, Zhang JS, Liu Q, Chen SY (2003). An EREBP/AP2-type protein in *Triticum aestivum* was a DRE-binding transcription factor induced by cold, dehydration and ABA stress. Theor Appl Genet.

[21] Zhang JZ, Creelman RA, Zhu JK (2004). From laboratory to field using information from *Arabidopsis* to engineer salt, cold, and drought tolerance in crops. Plant Physiol.

[22] Pennycooke JC, Cheng H, Roberts SM, Yang Q, Rhee SY, Stockinger EJ (2008). The low temperature-responsive, Solanum CBF1 genes maintain high identity in their upstream regions in a genomic environment undergoing gene duplications, deletions, and rearrangements. Plant Mol Biol.

[23] Pennycooke JC, Cheng H, Stockinger EJ (2008). Comparative genomic sequence and expression analyses of *Medicago truncatula* and alfalfa subspecies falcata COLD-ACCLIMATIONSPECIFIC genes. Plant Physiol.

[24] Soltesz A, Smedley M, Vashegyi I, Galiba G, Harwood W, Vagujfalvi A (2013). Transgenic barley lines prove the involvement of TaCBF14 and TaCBF15 in the cold acclimation process and in frost tolerance. J Exp Bot.

[25] Halliwell B (2007). Biochemistry of oxidative stress. Biochem Soc Trans.

[26] Hung SH, Yu CW, Lin CH (2005). Hydrogen peroxide functions as a stress signal in plants. Bot Bull Acad Sin.

[27] Mittler R, Vanderauwera S, Gollery M, Van BF (2004). Reactive oxygen gene network of plants. Trends Plant Sci.

[28] Dat J, Vandenabeele S, Vranova E, Van MM, Inze D, Van BF (2000). Dual action of the active oxygen species during plant stress responses. Cell Mol Life Sci.

[29] Zhu J, Dong CH, Zhu JK (2007). Interplay between cold-responsive gene regulation, metabolism and RNA processing during plant cold acclimation. Curr Opin Plant Biol.

[30] Knight H, Knight MR (2001). Abiotic stress signalling pathways: specificity and cross-talk. Trends Plant Sci.

[31] Davletova S, Schlauch K, Coutu J, Mittler R (2005). The zinc-finger protein Zat12 plays a central role in reactive oxygen and abiotic stress signaling in *Arabidopsis*. Plant Physiol.

[32] He F, Li HG, Wang JJ, Su YY, Wang HL, Feng CH, Yang YL, Niu MX, Liu C, Yin WL, Xia XL (2019). PeSTZ1, a C2H2-type zinc finger transcription factor from *Populus euphratica*, enhances freezing tolerance through modulation of ROS scavenging by directly regulating *PeAPX2*. Plant Biotechnol J.

[33] Liu MJ, Wang JR (2019). Fruit scientific research in new China in the past 70 years: Chinese jujube. J Fruit Sci.

[34] Gupta R, Deswal R (2014). Antifreeze proteins enable plants to survive in freezing conditions. J Biosci.

[35] Horton DE, Johnson NC, Singh D, Swain DL, Rajaratnam B, Diffenbaugh NS (2015). Contribution of changes in atmospheric circulation patterns to extreme temperature trends. Nature..

[36] Nasrabadi M, Ramezanian A, Eshghi S, KamgarHaghighi AA, Vazifeshenas MR, Valero D (2019). Biochemical changes and winter hardiness in pomegranate (*Punica granatum* L.) trees grown under deficit irrigation. Sci Hortic.

[37] Saadati S, Baninasab B, Mobli M, Gholami M (2019). Measurements of freezing tolerance and their relationship with some biochemical and physiological parameters in seven olive cultivars. Acta Physiol Plant.

[38] Niu RX, Zhao XM, Wang CB, Wang FL (2019). Transcriptome profiling of *Prunus persica* branches reveals candidate genes potentially involved in freezing tolerance. Sci Hortic.

[39] Shi YT, Ding YL, Yang SH (2018). Molecular regulation of CBF signaling in cold acclimation. Trends Plant Sci.

[40] Hong JH, Savina M, Du J, Devendran A, Ramakanth KK, Tian X, Sim WS, Mironova VV, Xu J (2017). A sacrifice-for-survival mechanism protects root stem cell niche from chilling stress. Cell..

[41] Zhang Z, Li J, Li F, Liu H, Yang W, Chong K, Xu Y (2017). OsMAPK3 phosphorylate OsbHLH002/OsICE1 and inhibits its ubiquitination to activate OsTPP1 and enhances rice chilling tolerance. Dev Cell.

[42] Ding YL, Jia Y, Shi YT, Zhang X, Song C, Gong Z, Yang S (2018). OST1-mediated BTF3L phosphorylation positively regulates CBFs during plant cold responses. EMBO J.

[43] Kou S, Chen L, Tu W, Federico S, Wang YM, Liu J, Fernie AR, Song B, Xie CH (2018). The arginine decarboxylase gene ADC1, associated to the putrescine pathway, plays an important role in potato cold-acclimated freezing tolerance as revealed by transcriptome and metabolome analyses. Plant J.

[44] Steponkus PL, Uemura M, Joseph RA, Gilmour SJ, Thomashow MF (1998). Mode of action of the COR15a gene on the freezing tolerance of *Arabidopsis thaliana*. Proc Natl Acad Sci.

[45] Kaplan F, Guy CL (2004). B-amylase induction and the protective role of maltose during temperature shock. Plant Physiol.

[46] Kaplan F, Guy CL (2005). RNA interference of Arabidopsis beta-amylase8 prevents maltose accumulation upon cold shock and increases sensitivity of PSII photochemical efficiency to freezing stress. Plant J.

[47] Kaplan F, Kopka J, Sung DY, Zhao W, Popp M, Porat R, Guy CL (2007). Transcript and metabolite profiling during cold acclimation of *Arabidopsis* reveals an intricate relationship of cold-regulated gene expression with modifications in metabolite content. Plant J.

[48] Dai N, Petreikov M, Portnoy V, Katzir N, Pharr DM, Schaffer AA (2006). Cloning and expression analysis of a UDP-galactose/glucose pyrophosphorylase from melon fruit provides evidence for the major metabolic pathway of galactose metabolism in raffinose oligosaccharide metabolizing plants. Plant Physiol.

[49] Kosovã K, Vitamvas P, Ilja P (2014). Wheat and barley dehydrins under cold, drought, and salinity-what can LEA-II protein tell us about plant stress response?. Front Plant Sci.

[50] Yang T, Chaudhuri S, Yang L, Du L, Poovaiah BW (2010). A calcium/calmodulin-regulated member of the receptor- like kinase family confers cold tolerance in plants. J Biol Chem.

[51] Tyystjarvi E (2013). Photoinhibition of photosystem II. Int Rev Cell Mol Biol.

[52] Heidarvand L, Maali-Amiri R (2013). Physio-biochemical and proteome analysis of chickpea in early phases of cold stress. J Plant Physiol.

[53] Qi JS, Song CP, Wang BS, Zhou JM, Kangasjarvi J, Zhu JK, Gong ZZ (2018). Reactive oxygen species signaling and stomatal movement in plant responses to drought stress and pathogen attack. J Integr Plant Biol.

[54] Lee SC, Lee MY, Kim SJ, Jun SH, An G, Kim SR (2005). Characterization of an abiotic stress inducible dehydrin gene, OsDhn1, in rice (*Oryza sativa* L). Mol Cells.

[55] Lee BH, Henderson DA, Zhu JK (2005). The *Arabidopsis* cold-responsive transcriptome and its regulation by ICE1. Plant Cell.

[56] Zhao C, Wang P, Si T, Hsu CC, Wang L, Zayed O, Yu Z, Zhu Y, Dong J, Tao WA, Zhu JK (2017). MAP kinase cascades regulate the cold response by modulating ICE1 protein stability. Dev Cell.

[57] Ding YL, Shi YT, Yang SH. Advances and challenges in uncovering cold tolerance regulatory mechanisms in plants. New Phytol. 2019. 10.1111/nph15696. 10.1111/nph.15696 30664232

[58] Li H, Ding Y, Shi Y, Zhang X, Zhang S, Gong Z, Yang S (2017). MPK3- and MPK6-mediated ICE1 phosphorylation negatively regulates ICE1 stability and freezing tolerance in *Arabidopsis*. Dev Cell.

[59] An JP, Li R, Qu FJ, You CX, Wang XF, Hao YJ (2018). An apple NAC transcription factor negatively regulates cold tolerance via CBF dependent pathway. J Plant Physiol.

[60] Fowler S, Thomashow MF (2002). *Arabidopsis* transcriptome profiling indicates that multiple regulatory pathways are activated during cold acclimation in addition to the CBF cold response pathway. Plant Cell.

[61] Vogel JT, Zarka DG, Van BA, Fowler SG, Thomashow MF (2005). Roles of the CBF2 and ZAT12 transcription factors in confi guring the low temperature transcriptome of *Arabidopsis*. Plant J.

[62] Xu Q, Wang WQ, Zeng JK, Zhang J, Grierson D, Li X, Yin XR, Chen KS (2015). A NAC transcription factor, EjNAC1, affects lignifcation of loquat fruit by regulating lignin. Postharvest Biol Technol.

[63] Zhong R, Lee C, Zhou J, McCarthy RL, Ye ZH (2008). A battery of transcription factors involved in the regulation of secondary cell wall biosynthesis in *Arabidopsis*. Plant Cell.

[64] Xie Z, Li D, Wang L, Sack FD, Grotewold E (2010). Role of the stomatal development regulators FLP/MYB88 in abiotic stress responses. Plant J.

[65] Xie YP, Chen PX, Yan Y, Bao C, Li XW, Wang LP, Shen XX, Li HY, Liu XF, Niu CD, Zhu C, Fang N, Shao Y, Zhao T, Yu JT, Zhu JH, Xu LF, Steven N, Ma FW, Guan QM (2018). An atypical R2R3 MYB transcription factor increases cold hardiness by CBF-dependent and CBF-independent pathways in apple. New Phytol.

[66] Vannini C, Locatelli F, Bracale M, Magnani E, Marsoni M, Osnato M, Mattana M, Baldoni E, Coraggio I (2004). Overexpression of the rice Osmyb4 gene increases chilling and freezing tolerance of *Arabidopsis thaliana* plants. Plant J.

[67] James AB, Syed NH, Bordage S, Marshall J, Nimmo GA, Jenkins GI, Herzyk P, Brown JWS, Nimmo HG (2012). Alternative splicing mediates responses of the *Arabidopsis* circadian clock to temperature changes. Plant Cell.

[68] Dong CH, Agarwal M, Zhang Y, Xie Q, Zhu JK (2006). The negative regulator of plant cold responses, HOS1, is a RING E3 ligase that mediates the ubiquitination and degradation of ICE1. Proc Natl Acad Sci USA.

[69] Wang WH, He EM, Guo Y, Tong QX, Zheng HL (2016). Chloroplast calcium and ROS signaling networks potentially facilitate the primed state for stomatal closure under multiple stresses. Environ Exp Bot.

[70] Kale AJ, Zonneveld AJ, Benes V, Berg MD, Koerkamp MG, Albermann K, Strack N, Ruijter JM, Richter A, Dujon B, Ansorge W, Tabak AHF, Botstein D (1999). Dynamics of gene expression revealed by comparison of serial analysis of gene expression transcript profiles from yeast grown on two different carbon sources. Mol Biol Cell.

[71] Eisen MB, Spellman PT, Brown PO, Botstein D (1998). Cluster analysis and display of genome-wide expression patterns. Proc Natl Acad Sci.

[72] Trapnell C, Pachter L, Salzberg SL (2009). TopHat: discovering splice junctions with RNA-Seq. Bioinformatics..

[73] Zhou HY, Jia JP, Kong DC, Zhang ZD, Song S, Li YY, Pang XM (2019). Genome-wide identification and analysis of the expression of DREB genes under abiotic stresses in Chinese jujube (*Ziziphus jujuba* mill.). J For Res.

