# Supplementary material for: Comparative transcriptome profiling reveals cold stress responsiveness in two contrasting Chinese jujube cultivars
Source: BMC Plant Biol. 2020 May 27;20:240. doi: 10.1186/s12870-020-02450-z (PMC7254757; doi:10.1186/s12870-020-02450-z)
Supplement: Supplementary file 1 — Additional file 1: Figure S1-S6 and Table S1-S2. Figure S1. The semi-lethal temperature (LT50, °C) of different cultivars after cold acclimation. Figure S2. Validation by qRT-PCR of DEGs isolated from the different samples in ‘Dongzao’ and ‘Jinsixiaozao’. Figure S3. GO analysis of DEGs under different degree cold stress in ‘Dongzao’. The X and Y axes correspond to GO terms and the number of DEGs. Figure S4. GO analysis of DEGs under different degree cold stress in ‘Jinsixiaozao’. The X and Y axes correspond to GO terms and the number of DEGs. Figure S5. DEGs in galactose metabolism. Different boxes represent different genes in galactose metabolism. The red boxes represent up-regulated genes, and the green boxes represents down-regulated genes. Figure S6. Alternative Splicing Event and gene number at the same degree cold stress between two cultivars. Table S1. Primers for qRT-PCR. Table S2. DEGs involved in galactose metabolism pathways. [file 12870_2020_2450_MOESM1_ESM.docx]

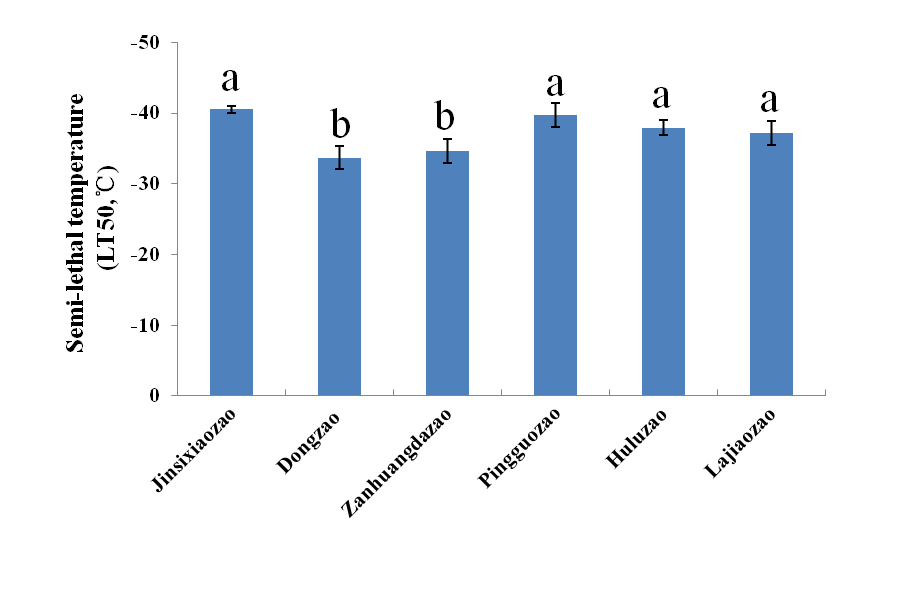


**Figure S1. The semi-lethal temperature (LT50, °C) of different cultivars after cold acclimation.** Different letters indicate significant difference between the cultivars (p < 0.05).


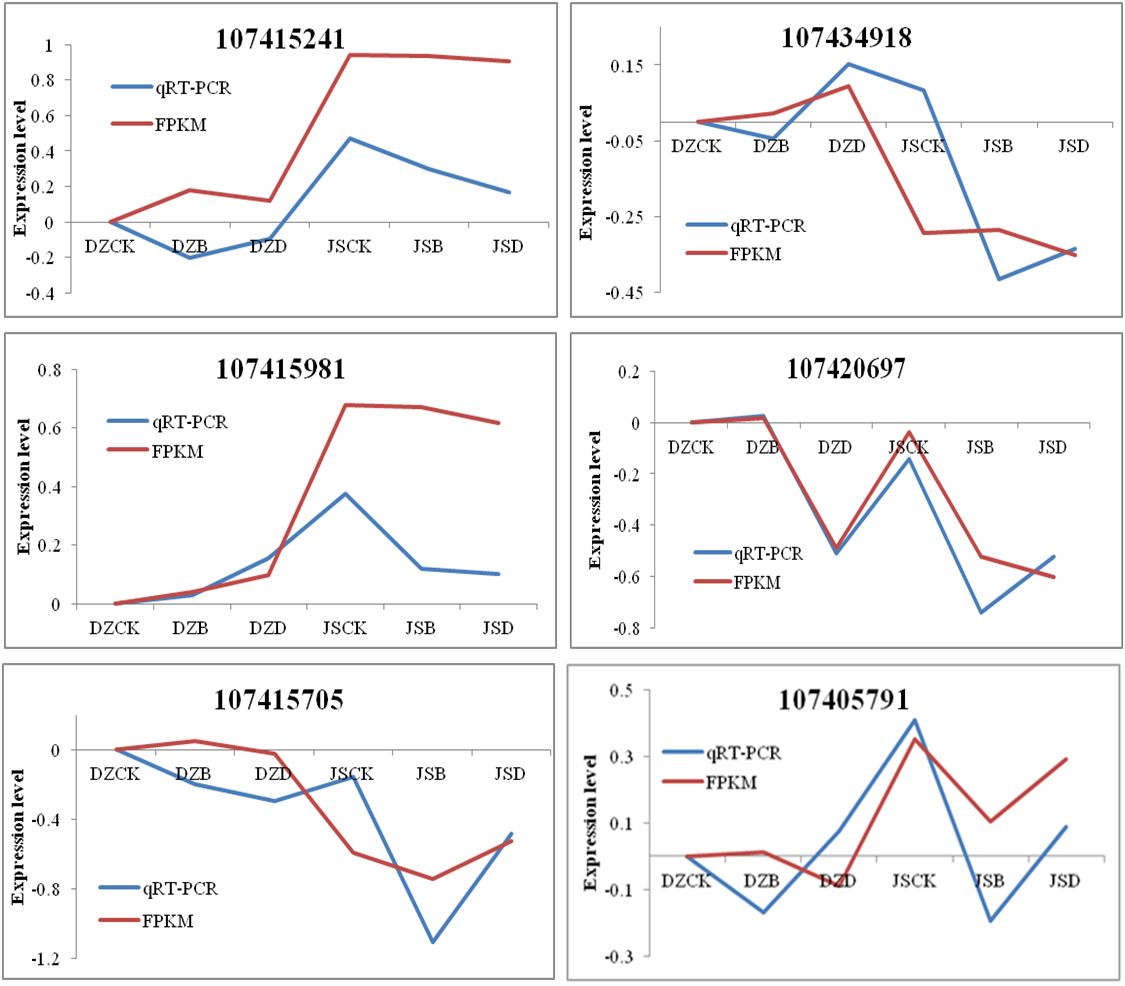


**Figure S2 Validation by qRT-PCR of DEGs isolated from the different samples in ‘Dongzao’ and ‘Jinsixiaozao’.** DZCK, ‘Dongzao’ treated at 4℃; DZA: ‘Dongzao’ treated at -10℃; DZB: ‘Dongzao’ treated at -20℃; DZC: ‘Dongzao’ treated at -30℃; DZD: ‘Dongzao’ treated at -40℃; JSCK: ‘Jinsixiaozao’ treated at 4℃; JSA: ‘Jinsixiaozao’ treated at -10℃; JSB: ‘Jinsixiaozao’ treated at -20℃; JSC: ‘Jinsixiaozao’ treated at -30℃; JSD: ‘Jinsixiaozao’ treated at -40℃.


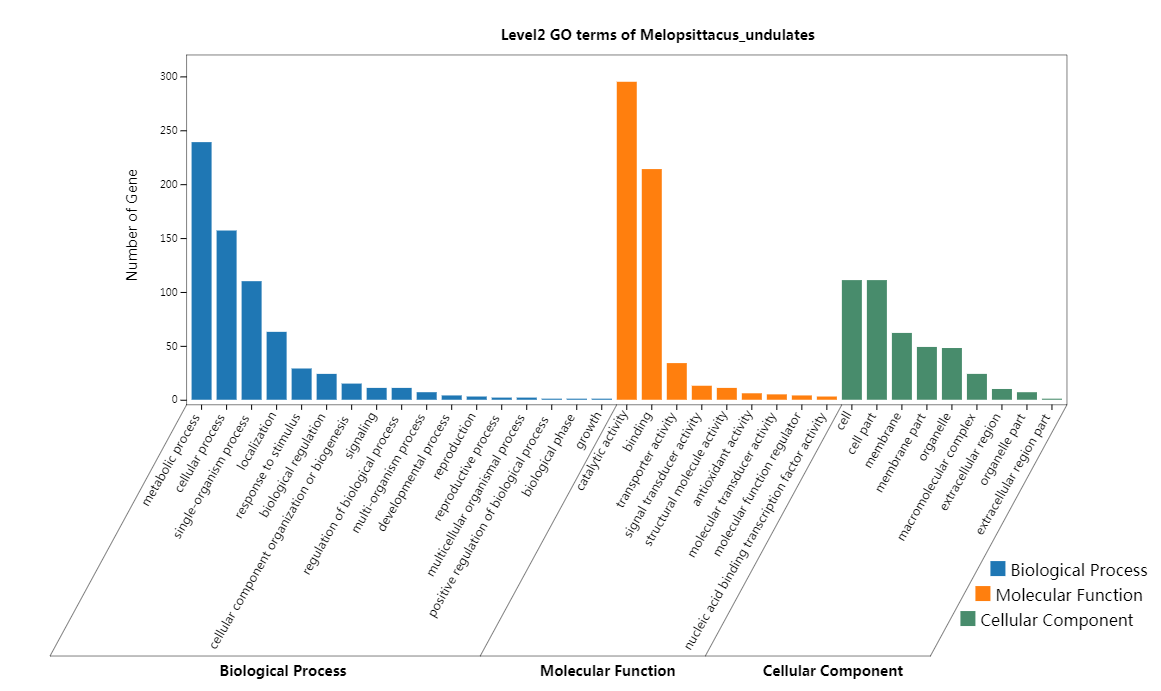


**Figure S3 GO analysis of DEGs under different degree cold stress in ‘Dongzao’.**

The X and Y axes correspond to GO terms and the number of DEGs.


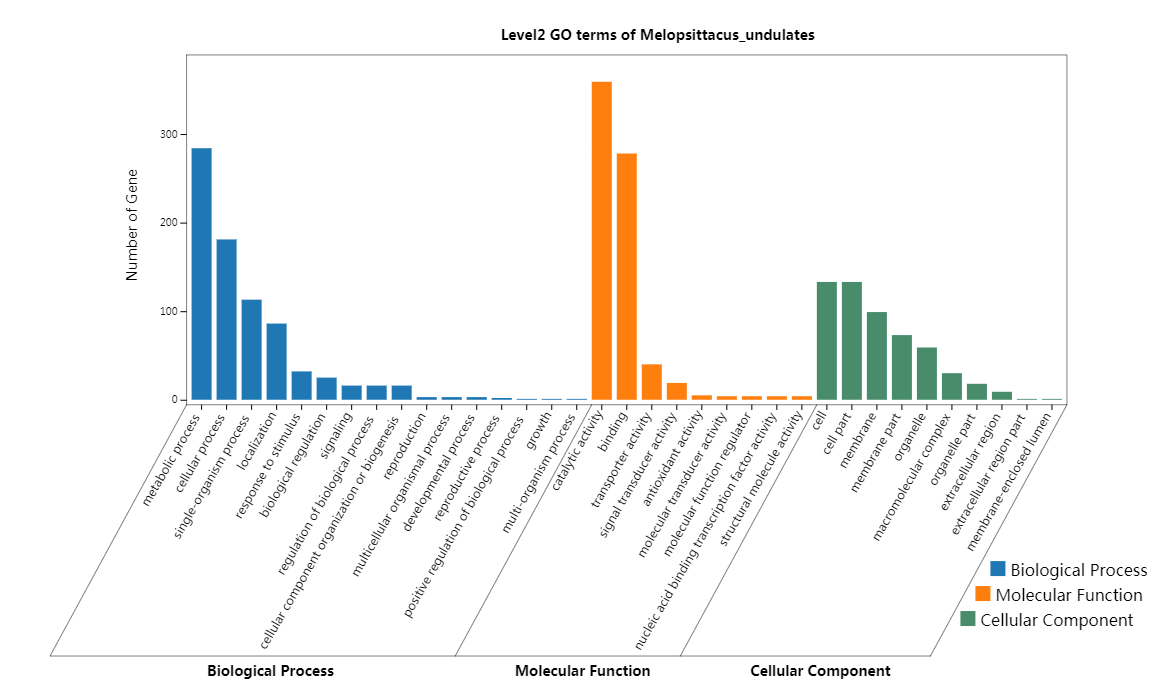


**Figure S4 GO analysis of DEGs under different degree cold stress in ‘Jinsixiaozao’.** The X and Y axes correspond to GO terms and the number of DEGs.


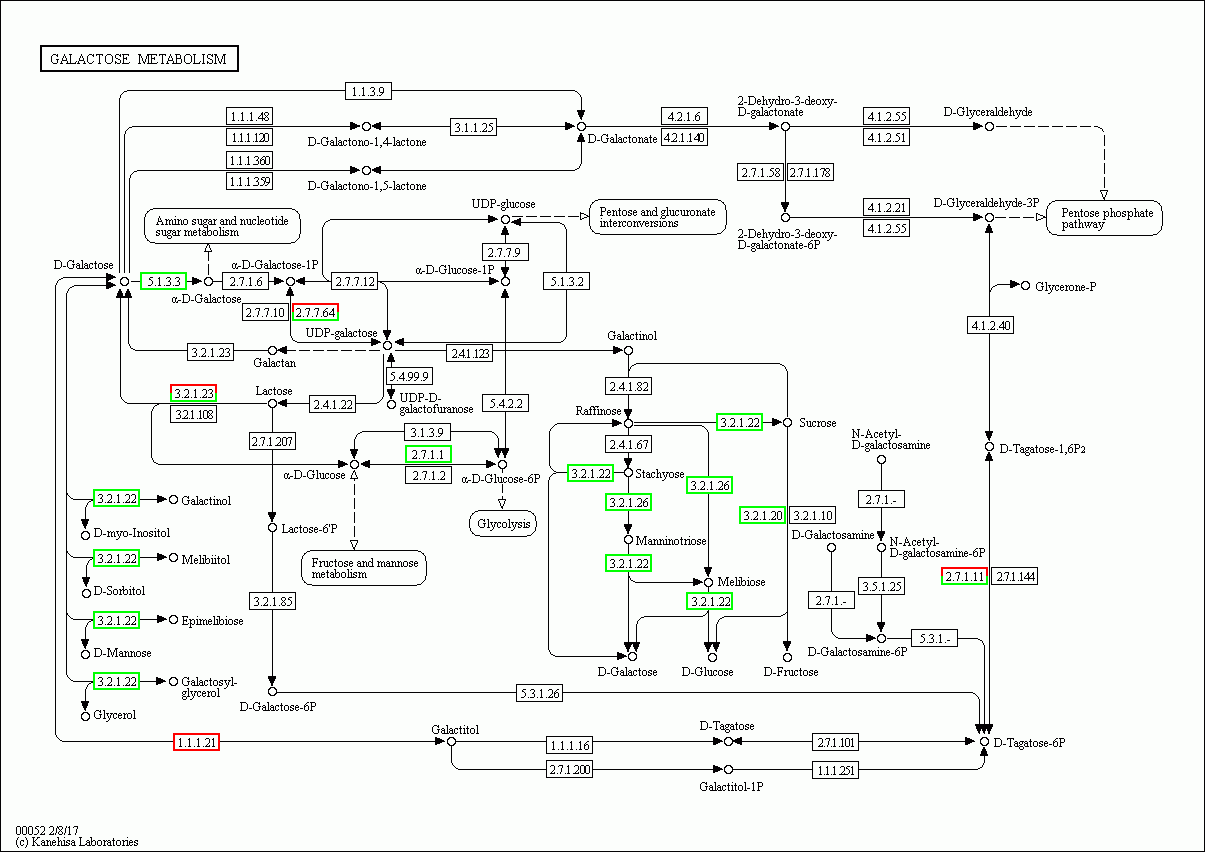


**Figure S5 DEGs in galactose metabolism pathways.**

Different boxes represent different genes in galactose metabolism. The red boxes represent up-regulated genes while the green boxes represent down-regulated genes.


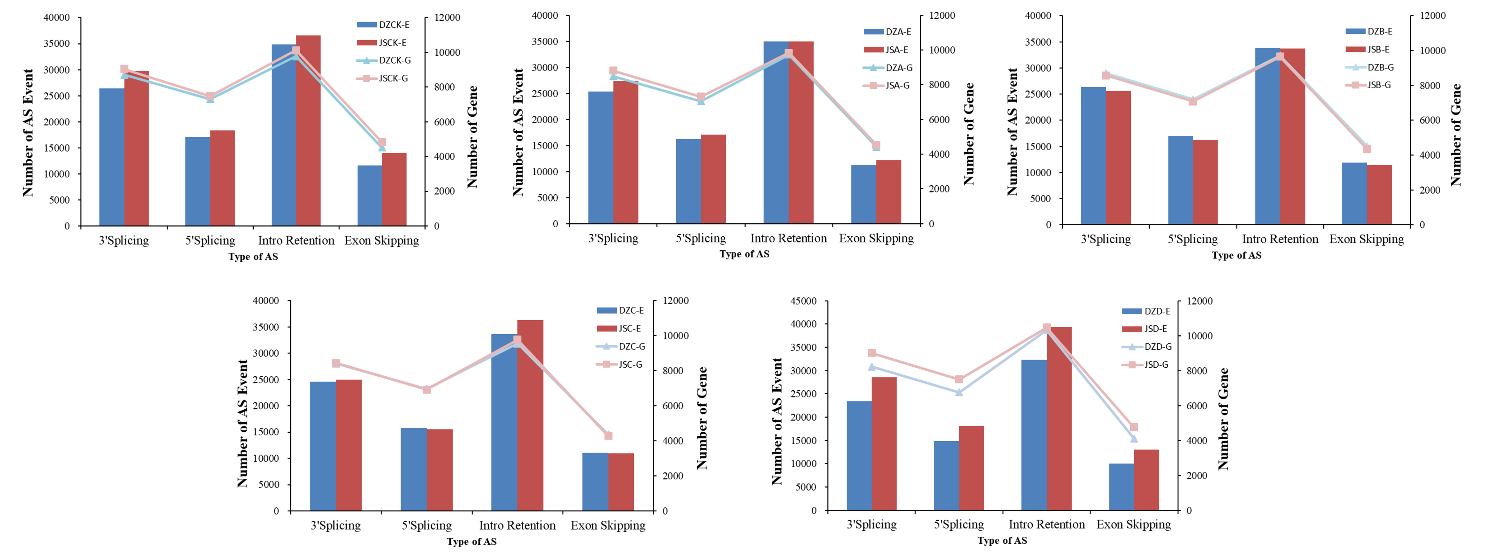


**Figure S6. Alternative Splicing Event and gene number at the same degree cold stress between two cultivars.** E, AS Event; G, Gene; DZCK, ‘Dongzao’ treated at 4℃; DZA: ‘Dongzao’ treated at -10℃; DZB: ‘Dongzao’ treated at -20℃; DZC: ‘Dongzao’ treated at -30℃; DZD: ‘Dongzao’ treated at -40℃; JSCK: ‘Jinsixiaozao’ treated at 4℃; JSA: ‘Jinsixiaozao’ treated at -10℃; JSB: ‘Jinsixiaozao’ treated at -20℃; JSC: ‘Jinsixiaozao’ treated at -30℃; JSD: ‘Jinsixiaozao’ treated at -40℃.

**Table S1 Primers for qRT-PCR.**

| Gene ID | Sequences 5'→3' | Annealing Temperature |
| --- | --- | --- |
| 107415241 | CAGACTCAGTGGTGGTATTGTG | 55^o^C |
|  | TGCTCATTCTCATCGGTAGGT |  |
| 107415981 | ATACACTAACCAAATCGCAACCA | 55^o^C |
|  | ACAGGACCAACAGAGGAAGAA |  |
| 107434918 | AACAACAACAACACTGGAGGTT | 55^o^C |
|  | TGGCACTGATTGAGAGGAAGA |  |
| 107420697 | CAGTCGTGATGGTGGTGAG | 55^o^C |
|  | TGGTGATGGTCCTGGTCTT |  |
| 107415705 | TCGTGCTATTGCTGCTAAGATAAC | 55^o^C |
|  | AACAAGTCACAGATGCCAACTC |  |
| 107405791 | AAGTCCAACACCGCCATTC | 55^o^C |
|  | GAGCAATTCACCGAGAGTCAA |  |
| *Zjactin* | GAGGAAGCAACTGGCAACTAAGG | 55^o^C |
|  | TACGAGCAAGCTGGATATCCTTC |  |

**Table S2 DEGs involved in galactose metabolism pathways.**

| Gene ID | Gene description | Pathway |
| --- | --- | --- |
| 107426117 | stachyose synthetase | Galactose metabolism;Metabolic pathways |
| 107415484 107417578 107418294 | raffinose synthase | Galactose metabolism;Metabolic pathways |
| 107411641 | inositol 3-alpha-galactosyltransferase | Galactose metabolism;Metabolic pathways |
| 107435337 107435723 | hexokinase | Glycolysis / Gluconeogenesis;Fructose and mannose metabolism; Galactose metabolism;Starch and sucrose metabolism; Amino sugar and nucleotide sugar metabolism; Streptomycin biosynthesis; Neomycin, kanamycin and gentamicin biosynthesis; Metabolic pathways; Biosynthesis of secondary metabolites;Microbial metabolism in diverse environments; Biosynthesis of antibiotics; Carbon metabolism; HIF-1 signaling pathway;Insulin signaling pathway;Type II diabetes mellitus;Carbohydrate digestion and absorption;Central carbon metabolism in cancer |
|  |  |  |
|  |  |  |
| 107408372 107406885 107411414 107408074 107407291 107408806 107407193 | 6-phosphofructokinase 1 | Glycolysis / Gluconeogenesis;Pentose phosphate pathway;Fructose and mannose metabolism; Galactose metabolism;Methane metabolism; Metabolic pathways; Biosynthesis of secondary metabolites; Microbial metabolism in diverse environments; Biosynthesis of antibiotics; Carbon metabolism; Biosynthesis of amino acids; RNA degradation; AMPK signaling pathway; Central carbon metabolism in cancer |
| 107409871 | UDPglucose--hexose-1-phosphate uridylyltransferase | Galactose metabolism;Amino sugar and nucleotide sugar metabolism;Metabolic pathways; Prolactin signaling pathway |
| 107421374 107418905 107404162 | UDP-sugar pyrophosphorylase | Pentose and glucuronate interconversions;Galactose metabolism Ascorbate and aldarate metabolism Amino sugar and nucleotide sugar metabolism Metabolic pathways Biosynthesis of antibiotics |
|  |  |  |
|  |  |  |
| 107430988 107418946 | maltase-glucoamylase | Galactose metabolism;Starch and sucrose metabolism;Metabolic pathways;Carbohydrate digestion and absorption |
| 107414011 107428511 | alpha-galactosidase | Galactose metabolism;Glycerolipid metabolism;Sphingolipid metabolism;Glycosphingolipid biosynthesis - globo and isoglobo series;Metabolic pathways;Lysosome |
| 107424008 107417196 107426293 107420256 107409426 107403993 107428314 107425187 107425355 107425261 | beta-galactosidase | Galactose metabolism;Other glycan degradation;Sphingolipid metabolism; Metabolic pathways |
|  |  |  |
| 107425264 107430302 | beta-fructofuranosidase | Galactose metabolism;Starch and sucrose metabolism;Metabolic pathways |
| 107419312 | aldose 1-epimerase | Glycolysis / Gluconeogenesis;Galactose metabolism; Metabolic pathways; Biosynthesis of secondary metabolites; Microbial metabolism in diverse environments;Biosynthesis of antibiotics |
